# Antiviral Activity of Halogenated Compounds Derived from L-Tyrosine Against SARS-CoV-2

**DOI:** 10.3390/molecules30071419

**Published:** 2025-03-22

**Authors:** Paula A. Velásquez-Bedoya, María I. Zapata-Cardona, Laura M. Monsalve-Escudero, Jaime A. Pereañez, Diego Guerra-Arias, Manuel Pastrana-Restrepo, Elkin Galeano, Wildeman Zapata-Builes

**Affiliations:** 1 Grupo Infettare, Facultad de Medicina, Universidad Cooperativa de Colombia, Calle 50 # 40-74, Medellín 050001, Colombia; pau.velasquez1501@gmail.com; 2 Grupo Inmunovirología, Facultad de Medicina, Universidad de Antioquia UdeA, Medellín 050001, Colombia; mariaisab5@gmail.com (M.I.Z.-C.); lmilena.monsalve@udea.edu.co (L.M.M.-E.); 3 Grupo de Investigación Promoción y Prevención Farmacéutica, Facultad de Ciencias Farmacéuticas y Alimentarias, Universidad de Antioquia UdeA, Medellín 050001, Colombia; jaime.pereanez@udea.edu.co; 4 Instituto de Parasitología y Biomedicina “López-Neyra”, Consejo Superior de Investigaciones Científicas, PTS Granada, 18016 Granada, Spain; diegoguerraar@gmail.com; 5 Programa de Estudio y Control de Enfermedades Tropicales PECET, Facultad de Medicina, Universidad de Antioquia, Medellín 050010, Colombia; 6 Grupo Productos Naturales Marinos, Departamento de Farmacia, Facultad de Ciencias Farmacéuticas y Alimentarias, Universidad de Antioquia UdeA, Medellín 050001, Colombia; mhpr2017@gmail.com (M.P.-R.); elkin.galeano@udea.edu.co (E.G.)

**Keywords:** SARS-CoV-2, COVID-19, antiviral activity, L-tyrosine, halogenated compounds

## Abstract

Introduction: Currently, there are no effective medications for treating all the clinical conditions of patients with COVID-19. We aimed to evaluate the antiviral activity of compounds derived from L-tyrosine against the B.1 lineage of SARS-CoV-2 in vitro and in silico. Methodology: The cytotoxicities of 15 halogenated compounds derived from L-tyrosine were evaluated in Vero-E6 cells by the MTT assay. The antiviral activity of the compounds was evaluated using four strategies, and viral quantification was performed by a plaque assay and qRT-PCR. The toxicity of the compounds was evaluated by ADMET predictor software. The affinity of these compounds for viral or cellular proteins and the stability of their conformations were determined by docking and molecular dynamics, respectively. Results: TODC-3M, TODI-2M, and YODC-3M reduced the viral titer >40% and inhibited the replication of viral RNA without significant cytotoxicity. In silico analyses revealed that these compounds presented low toxicity and binding energies between −4.3 and −5.2 Kcal/mol for three viral proteins (spike, Mpro, and RdRp). TODC-3M and YODC-3M presented the most stable conformations with the evaluated proteins. Conclusions: The most promising compounds were TODC-3M, TODI-2M, and YODC-3M, which presented low in vitro and in silico toxicity, antiviral potential through different strategies, and favorable affinities for viral targets. Therefore, they are candidates for in vivo studies.

## 1. Introduction

Coronavirus disease 2019 (COVID-19), caused by severe acute respiratory syndrome coronavirus 2 (SARS-CoV-2), was first reported in December 2019 by the Program for Monitoring Emerging Diseases in Wuhan, China [1]. In March 2020, COVID-19 was declared a global pandemic, and to date, more than 774 million infected people and more than 7 million deaths have been reported worldwide [1,2]. The clinical frame of patients infected with SARS-CoV-2 ranges from asymptomatic patients to mild or severe patients [3]. The factors that increase the risk of serious symptoms from COVID-19 include age, an immunocompromised or weakened immune system, and underlying health conditions such as obesity, pulmonary disorders, or heart disease [4].

Different developed vaccines have demonstrated efficacy in reducing the mortality rate caused by the disease. However, some studies have shown that their effectiveness against infections, hospitalizations, and mortality in patients with COVID-19 decreases over time [5,6]. Furthermore, they result in reduced immunity in immunocompromised patients [7], and their effectiveness is lower against circulating variants such as Omicron [8,9]. In addition, several serious adverse effects, such as myocarditis, anaphylactic symptoms, adenopathy, and thrombocytopenic thrombosis syndrome, have been reported for these vaccines [10].

On the other hand, to help prevent severe illness or death in patients with COVID-19, antiviral treatments have been used to inhibit specific steps of viral replication; the most significant treatments approved include nirmatrelvir, remdesivir, and molnupiravir [11]. Nirmatrelvir inhibits the main protease of SARS-CoV-2 (Mpro, also referred to as 3CLpro), whereas remdesivir and molnupiravir interfere with viral RNA replication through binding to RdRp (RNA-dependent RNA polymerase) or inducing viral mutagenesis by the incorporation into viral RNA strands, respectively [11]. These drugs have been approved for the treatment of COVID-19 in adults and pediatric patients with different clinical conditions, hospitalization statuses, or risks of severe disease [12]. However, they may have interactions [13,14] or adverse effects (headache, gastrointestinal symptoms, alteration of liver enzymes, coagulation, blood pressure, or hypersensitivity, among others), costs, or administration routes that make them less accessible to the general population [15,16,17]. Furthermore, some of them are not recommended for lactating or pregnant women [18].

Considering the limitations of available treatments and vaccines, that there are no approved drugs to reduce the spread of the virus, and that the constant emergence of SARS-CoV-2 variants can increase the number of infected individuals and decrease the effectiveness of vaccines, it is necessary to continue the search for new therapeutic strategies through second-use drugs or new bioactive compounds against SARS-CoV-2.

In this context, bioactive compounds are those with biological activity that directly affects a living organism and can provide health benefits. These are produced by plants, fungi, and animals, among which are marine sponges that produce them as a defense mechanism. Some of the compounds identified in marine sponges are terpenes, sterols, unusual nucleosides, cyclic peptides, fatty acids, peroxides, and amino acid-derivative halogenated molecules [19]. 

Multiple biological activities have been reported for the halogenated compounds, including antifungal, insecticidal, antiparasitic, antibacterial, antiviral, anti-inflammatory, and antiproliferative activities [20,21]. In addition, studies have related the incorporation of halogen substituents with an enhancement in the metabolic stability, lipophilicity, and electronegativity, which may confer advantages to the biophysical and -chemical properties of the compounds [22]. Further, these molecules can form halogen bonds similar to hydrogen bonds, which can increase the affinity with target proteins and may contribute to controlling different diseases [23]. 

With respect to antiviral activity, the halogenated compounds have demonstrated potential against viruses such as human immunodeficiency virus 1 (HIV-1), Zika (ZIKV), Chikungunya (CHIKV), hepatitis E (HEV), Rift Valley river virus (RVFV), SARS-CoV-2, and human coronavirus NL63 (HCoV-NL63) [19,22,24,25]. Different mechanisms have been proposed, including the inhibition of viral RNA, the interaction with non-structural proteins evolving in the replication or processing of polyproteins, or affinity by viral envelope proteins [24,25,26].

Previously, Galeano et al. isolated compounds derived from L-tyrosine, dihalogenated and methylated in their amino or hydroxyl groups from sponges of the Gulf of Urabá (Colombia) of the order Verongida [27]. For economic reasons and protection of natural resources, these compounds were later synthesized in the laboratory to evaluate their biological activity. This study assessed the in vitro antiviral activity of 15 derivative compounds from L-tyrosine against an ancestral strain (lineage B.1) of SARS-CoV-2, using four antiviral strategies. In silico studies were also performed to suggest the affinity and stability of the interactions of these compounds with three viral proteins (spike or S, Mpro, and RdRp) and the cell receptor ACE2 (angiotensin-converting enzyme 2) by two bioinformatic tools (molecular docking and molecular dynamics).

## 2. Results

### 2.1. Halogenated Compounds Derived from L-Tyrosine Do Not Have Significant Cytotoxic Effects on Vero-E6 Cells

**Figure 1:**
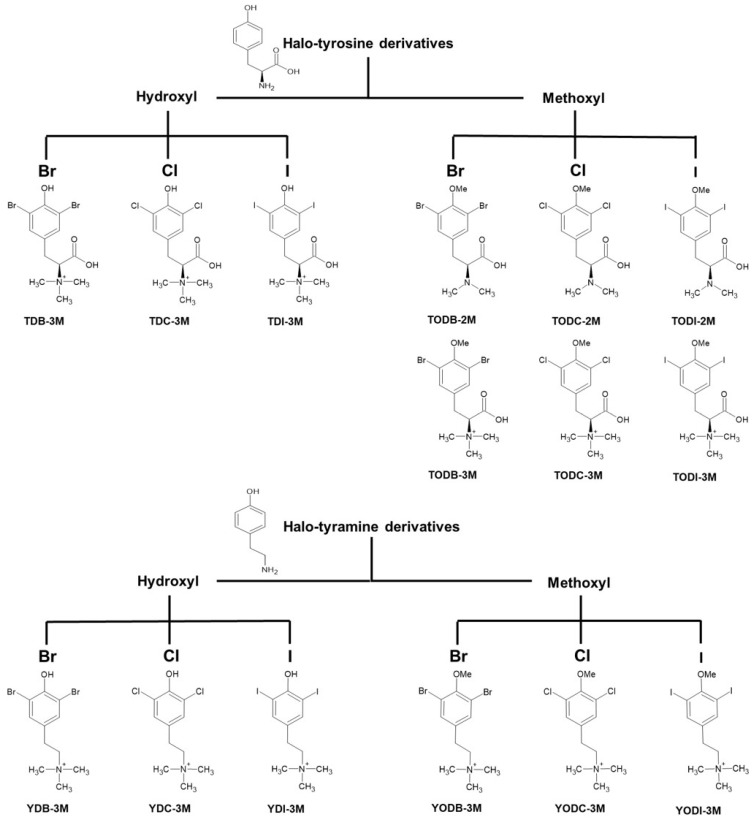
Classification of halogenated compounds derived from L-tyrosine. Fifteen halogenated compounds derived from L-tyrosine were evaluated and classified into halotyrosine and halotyramine derivatives. These groups were further subdivided into compounds with free phenolic OH and methylated phenolic OH (methyl ether derivatives). Finally, each subgroup was disrupted into compounds classified by halogen type (bromine [Br], chlorine [Cl], and iodine [I]) and by the number of methylations in the amino group.

**Figure 2:**
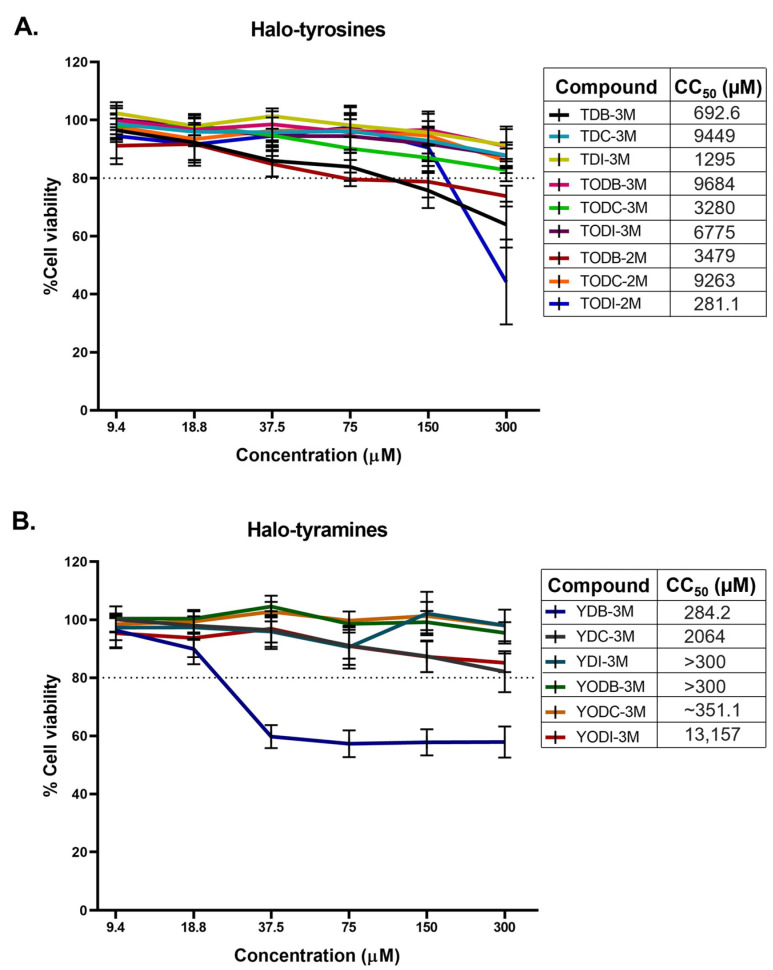
Halogenated compounds derived from L-tyrosine did not have significant cytotoxic effects on Vero-E6 cells. Vero-E6 cells were treated with different concentrations (from 9.4 μM to 300 μM for 48 h) of halotyrosine derivative compounds (**A**) and halotyramine derivative (**B**) compounds. The percentage of viability (% cell viability) was calculated relative to a control of cells without treatment (control of cell viability). The results are shown as the mean ± standard deviation (SD), n = 8. The dotted line indicates 80% cell viability. The CC50 (50% cytotoxic concentration) values for each compound were included.

Fifteen halogenated compounds classified into halotyrosine and halotyramine derivatives were included in this study (Figure 1). The toxicity of the compounds on Vero-E6 cells was determined by the MTT (3-(4,5-Dimethylthiazol-2-yl)-2,5-diphenyltetrazolium bromide) assay. Among the nine halotyrosine derivatives evaluated, only TDB-3M, TODB-2M, and TODI-2M showed >20% cytotoxicity in these cells at concentrations higher than 75, 37.5, and 150 µM, respectively (Figure 2A). Among the six halotyramine derivatives, only YDB-3M was cytotoxic at concentrations higher than 18.8 µM (Figure 2B). The other compounds did not show significant cytotoxicity at the concentrations evaluated (9.4-300 µM). The compounds with the lowest toxicity were TDI-3M, TODB-3M, YDI-3M, YODB-M, and YODC-3M. The CC50 (50% cytotoxic concentration) values for each treatment are shown in front of each compound. The solvent (DMSO) of the compounds was not cytotoxic at the concentrations used (Figure S1A). Moreover, chloroquine (CQ), the positive control of viral inhibition, at 50 µM and 100 µM did not affect cell viability (Figure S1B).

### 2.2. Two Halotyrosine and Three Halotyramine Derivatives Reduce the Viral Titer of SARS-CoV-2

**Figure 3:**
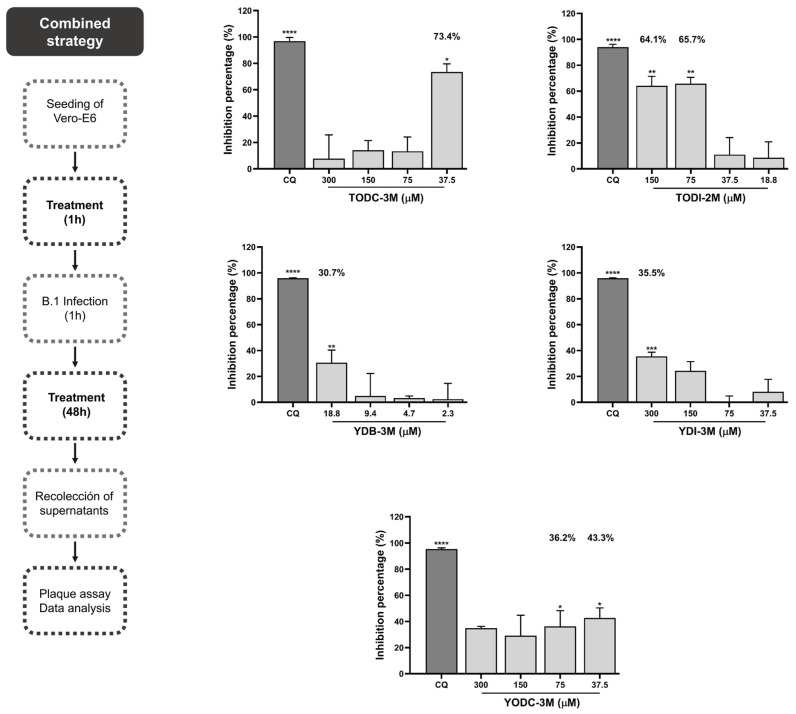
The halotyrosine derivatives TODC-3M and TODI-2M and the halotyramine derivatives YDB-3M, YDI-3M, and YODC-3M reduced the viral titer of SARS-CoV-2. The viral titers (PFU/mL) obtained after combined strategy with the five halogenated compounds were quantified. The figure shows the inhibition percentages calculated from the comparison of viral titers of each treatment with respect to an untreated control (control of infection without treatment). Chloroquine (CQ) was used as a positive control of viral inhibition (50 μM). Four replicates and two experimental units were used for each concentration. Significance was calculated according to the normality of the data and with respect to the untreated control. Symbol meaning: * *p* ≤ 0.05, ** *p* ≤ 0.01, *** *p* ≤ 0.001, **** *p* ≤ 0.0001.

The antiviral effect of the compounds against the lineage B.1 of SARS-CoV-2 was evaluated by a combined strategy in Vero-E6 cells. Two halotyrosine derivatives (TODC-3M and TODI-2M) significantly reduced viral infectivity. TODC-3M reduced the viral titer by 73.4% at 37.5 µM. The IC50 (half maximal inhibitory concentration) for this compound was 47.1 µM, and the calculated SI (selectivity index) was 69.6. TODI-2M significantly reduced the viral titer by 64.1% and 65.7% at 150 and 75 µM, respectively (IC50 = 90.1 µM, SI = 3.1). The halotyramine derivatives YDB-3M (30.7% at 18.8 µM), YDI-3M (35.5% at 300 µM), and YODC-3M (36.2% and 43.3% at 75 and 37.5 µM, respectively) significantly reduced the viral titer by this antiviral strategy (Figure 3). The IC50s calculated for YDB-3M, YDI-3M, and YODC-3M were 31.3, 479.9, and 18.8 µM, respectively, and the SI values were 9.1, ~0.6, and ~18.7, respectively.

### 2.3. TODC-3M, TODI-2M, and YODC-3M Showed Significant Effectiveness Against SARS-CoV-2 Through Individual Strategies

**Figure 4:**
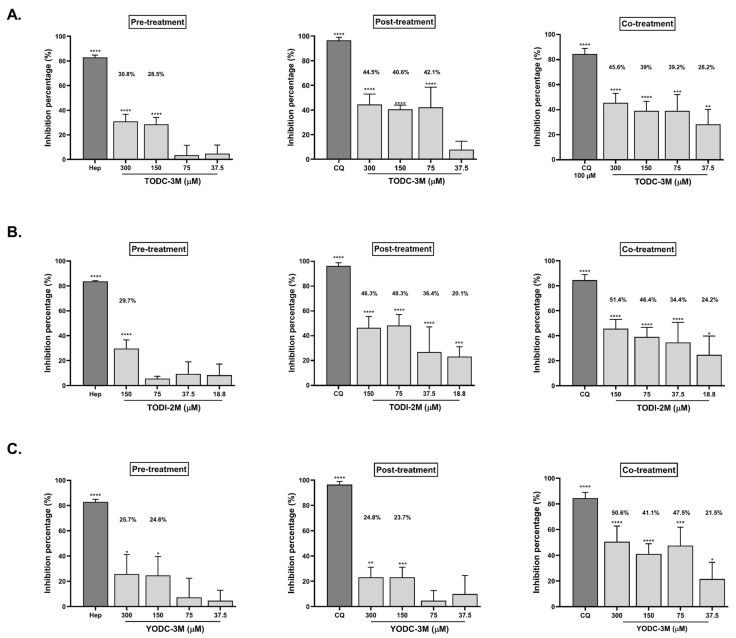
TODC-3M, TODI-2M, and YODC-3M showed significant effectiveness against SARS-CoV-2 through individual strategies. The viral titers after antiviral evaluation of TODC-3M (**A**), TODI-2M (**B**), and YODC-3M (**C**) using three strategies (pre-, post-, and co-treatment) were quantified by plaque assay. The bars show the inhibition percentages calculated by comparing each treatment with an untreated control (control of infection without treatment). Heparin (50 µM) was selected as a positive control of viral inhibition in pre-treatment, while CQ was used for post-treatment and co-treatment (50 µM and 100 μM, respectively). The significance was calculated according to the data distribution and concerning an untreated control. * *p* ≤ 0.05, ** *p* ≤ 0.01, *** *p* ≤ 0.001, **** *p* ≤ 0.0001.

Among the five compounds with antiviral activity, those that reduced the viral titer by up to 40% (TODC-3M, TODI-2M, and YODC-3M) were considered as promising and evaluated through three individual strategies: pre-, post-, and co-treatment (Figure 4).

TODC-3M, TODI-2M, and YODC-3M significantly reduced the viral titer under all the antiviral strategies. TODC-3M was promising for post-treatment, where the inhibition rates ranged from 40.6% to 44.5% at concentrations between 75 and 300 μM. This compound also inhibited infection by co-treatment (45.6% at 300 µM) (Figure 4A). In both treatment strategies, the higher viral inhibition was observed at the maximum concentration evaluated of TODC-3M (300 µM). TODI-2M exhibited inhibition percentages of up to 48.3% and 51.4% by post-treatment and co-treatment, respectively (Figure 4B), with a reduction depending on the dose. Finally, co-treatment with YODC-3M decreased the viral titer by 41.1% to 50.6% at concentrations greater than or equal to 75 µM (Figure 4C).

### 2.4. Promising Compounds Inhibit SARS-CoV-2 RNA Replication

**Figure 5:**
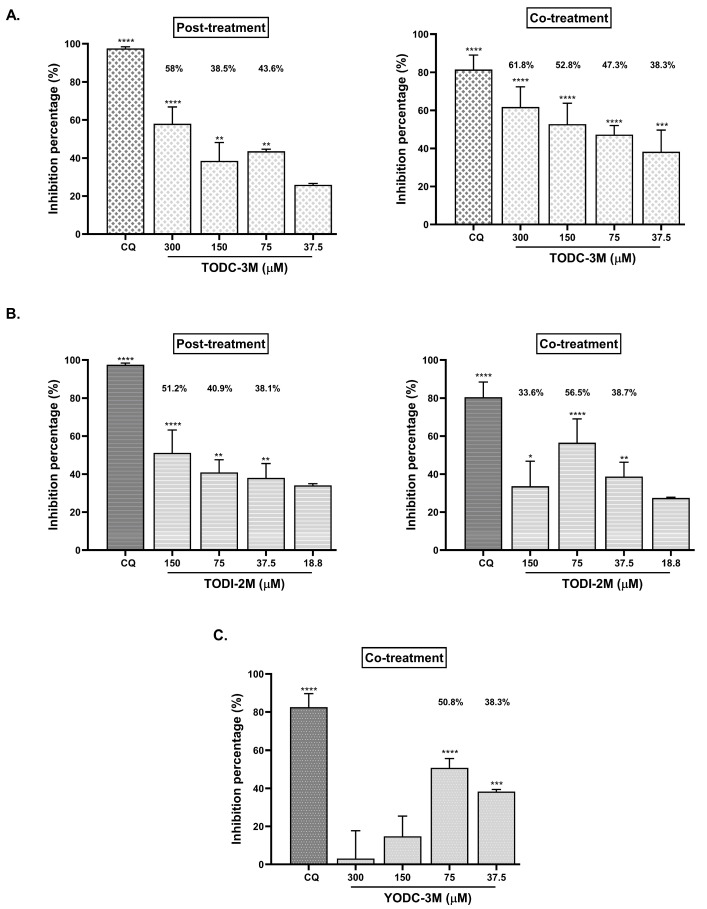
TODC-3M, TODI-2M, and YODC-3M reduced the amount of intracellular SARS-CoV-2 RNA when individual strategies were used. Vero-E6 monolayers infected with lineage B.1 of SARS-CoV-2 and treated with TODC-3M (**A**) and TODI-2M (**B**) by post- and co-treatment, and with YODC-3M through co-treatment (**C**), were used to quantify the number of copies of the SARS-CoV-2 E gene by qRT-PCR. The bars show the percentages of inhibition of the intracellular E gene after each treatment in comparison with an untreated control (monolayers infected without treatment). The positive control of viral inhibition used for post- and co-treatment was CQ (50 µM and 100 µM, respectively). Significances were calculated according to the data normality. Symbol meaning: * *p* ≤ 0.05, ** *p* ≤ 0.01, *** *p* ≤ 0.001, **** *p* ≤ 0.0001 with respect to an untreated control.

The monolayers treated with promising compounds by individual strategies were analyzed by real-time quantitative reverse transcription PCR (qRT-PCR) (Figure 5). TODC-3M significantly reduced SARS-CoV-2 RNA through post-treatment at 75, 150, and 300 μM, with decreases of 43.6%, 38.5%, and 58%, respectively. This reduction was also observed with the co-treatment strategy, with inhibition percentages ranging from 38.3–61.8% at all the evaluated concentrations (37.5–300 μM). The TODI-2M compound significantly inhibited viral RNA through post-treatment and co-treatment at concentrations ranging from 37.5 to 150 μM (percentages up 51.2% and 56.5% by post-treatment and co-treatment, respectively). On the other hand, YODC-3M significantly reduced the amount of intracellular viral RNA produced by co-treatment (38.3% and 50.8% at 37.5 µM and 75 µM, respectively) (Figure 5).

### 2.5. According to In Silico Predictions, the Compounds Presented a Low Probability of Toxicity in Organs and Tissues

Due to the low toxicity observed in the in vitro assays, we used the ADMET Predictor® v8 software (Simulations Plus, Inc. Lancaster, CA, USA) to identify biological toxicity risks associated with the use of the 15 halogenated compounds. 

The in silico toxicity predictions for all the compounds and commercial drugs are described in Table 1. According to the risk score, the promising compounds had a lower risk score than the commercial drugs used as controls. In this sense, the risk scores for TODC-3M, TODI-2M, and YODC-3M were 1.0, 0.5, and 1.9, respectively, which indicates a lower probability of presenting toxic effects in vivo.

It is important to mention that TODC-3M and TODI-2M can cause endocrine toxicity (by binding to the active site of estrogen or androgen receptors), chromosomal aberrations, reproductive toxicity, or liver toxicity through increases in the GGT (gamma-glutamyl transferase). YODC-3M may cause respiratory sensitization, cardiac toxicity (due to binding to specific potassium channels in the heart), neuronal toxicity (due to the generation of phospholipidosis that can alter neuronal signaling) and endocrine toxicity (Table 1).

Additionally, a cluster analysis (nearest neighbor method) carried out in Statgraphics® 19 centurion (Statgraphics Technologies. The Plains, VA, USA), through which the effectiveness (IC50) of the compounds and toxicity (CC50 and ADMET risk score) were related, showed the grouping of the compounds and controls into three large groups (Figure S2). All the compounds with in vitro antiviral activity against SARS-CoV-2 in at least one strategy were included in the first cluster, the commercial medications were in the second cluster, and the halogenated compounds without in vitro antiviral activity were included in the third cluster. Interestingly, the promising compound with the best selectivity (TODC-3M) was found in one of the groups closest to the commercially used drug group (Figure S2).

### 2.6. In Silico Analyses of Compounds with Antiviral Activity Revealed Favorable Binding Energies Against Viral and Cellular Proteins

**Figure 6:**
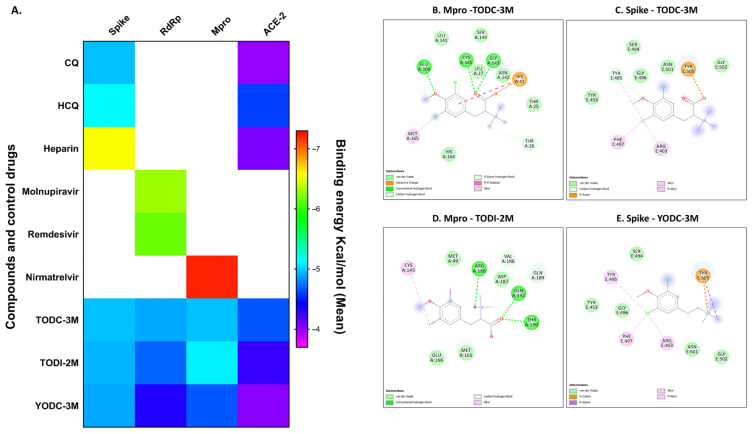
In silico interactions between halogenated compounds and viral and cellular proteins. The figure shows the heatmap of the binding free energies between the ligands with three SARS-CoV-2 proteins (spike, RdRp, Mpro) and the cellular protein ACE2. Data were obtained from a molecular docking analysis using AutodockVina®. Three docking simulations with the same settings for each complex were performed, and the average was plotted on a color scale (**A**). The 2D visualization between ligands and target proteins is shown. The spheres represent the interacting amino acids in the following complexes: Mpro-TODC-3M (**B**)**,** Spike-TODC-3M (**C**), Mpro-TODI-2M (**D**), and Spike-YODC-3M (**E**). The amino acids of the spike protein with a blue border are those that interact with the ACE2 receptor. For Mpro, they represent the amino acids associated with their catalytic function. Discovery Studio was used for visualization of the complexes.

To suggest a possible mode of action of the promising compounds a molecular docking was performed. The interactions of TODC-3M, TODI-2M, and YODC-3M with three SARS-CoV-2 proteins (spike, RdRp, and Mpro) and the cellular protein ACE2 were evaluated. All ligands presented favorable binding energies for the proteins evaluated, mainly for spike and Mpro (Figure 6A).

The best binding energy of TODC-3M was for the spike and Mpro proteins (−5.0 Kcal/mol for both cases). The TODI-2M ligand displayed better binding energy for the Mpro protein, with a value of −5.2 Kcal/mol. Finally, the Spike-YODC-3M complex presented a value of −4.9 Kcal/mol (Figure 6A).

The controls of interaction used for the S protein were CQ (−5.0 Kcal/mol) and HCQ (−5.1 Kcal/mol); for RdRp, they were molnupiravir (−6.3 Kcal/mol) and remdesivir (−6.2 Kcal/mol); for Mpro, they were nirmatrelvir (−7.2 Kcal/mol); and for ACE2, they were CQ (−4.0 Kcal/mol) and HCQ (−4.6 Kcal/mol). Interestingly, some of these controls presented lower binding energies than did the ligands used by the proteins, which indicates that these interactions can be generated more efficiently (Figure 6A).

The molecular interactions of the previous compounds with the spike and Mpro proteins of SARS-CoV-2 were visualized in 2D and 3D (Figure 6 and Figure S3). TODC-3M interacted with two amino acids responsible for the proteolytic function of Mpro (Cys145 and His41) [28]. This ligand formed conventional and Pi-donor Hydrogen bonds with Cys145 and it has an attractive charge and a Pi-Pi Stacked interaction with His41 (Figure 6B). In addition, TODC-3M interacted with Tyr453, Gly496, Ser494, Gly502, and Tyr505 of the S protein (Figure 6C), which are responsible for the attachment of the virus to the cell [28,29]. TODI-2M formed three hydrogen bonds, four van der Waals interactions, and two carbon hydrogen bridges with the amino acids of the active site of Mpro (Met49, Met165, Glu166, Val186, Asp187, Arg188, Gln189, Thr190, and Gln192), as well as an alkyl-type interaction with the catalytic amino acid Cys145 (Figure 6D). Finally, the halotyramine YODC-3M formed interactions type pi–cation, Pi-Sigma, Alkyl, Pi-Alkyl and van der Waals interactions with amino acids participate in the binding of the S protein to the ACE2 receptor (Figure 6E). Table 2 summarizes all the in vitro and in silico results of the promising compounds with antiviral activity mentioned above.

### 2.7. Molecular Dynamics Simulation of the Compounds with the Tested Proteins

To assess the potential stability between ligands and target proteins at the docking position, a series of molecular dynamics simulations were performed. The RMSD (root mean squared deviation), principal component analysis (PCA), free energy landscape (FEL), and clustering of the complex were used to monitor how the position of the ligand changed during the simulation.

**Figure 7:**
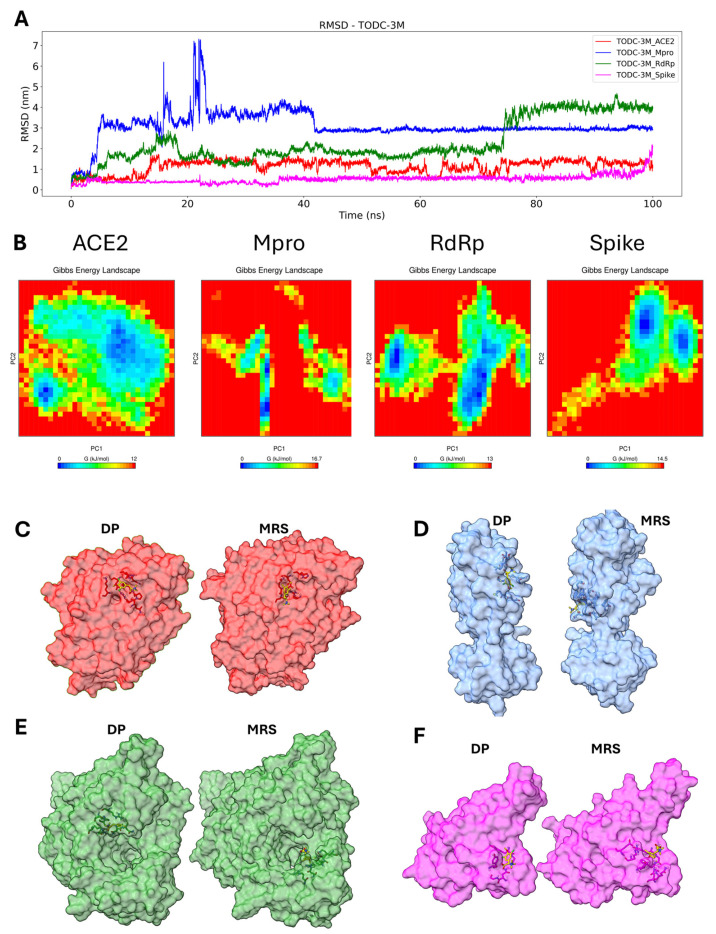
Molecular dynamics analysis of the TODC-3M ligand with the target proteins. The RMSD is from the molecular dynamic trajectories for TODC-3M, ACE2 (red), Mpro (blue), RdRp (green), and spike (magenta) (**A**). PCA-based FEL of the complex; blue indicates the minimum energy (stable conformation), and red indicates the maximum energy (unstable conformation) (**B**). Clustering of the more representative conformations during 100 ns: DP, MRS, ACE2 (**C**), Mpro (**D**), RdRp (**E**), spike (**F**). TODC-3M is represented in yellow.

The TODC-3M ligand only preserved its initial position with the spike protein for 100 ns of simulation; for the other three proteins, the initial conformation was not stable enough to maintain the docking position (DP) during the simulation, prompting the compound to search for a more stable pocket in the proteins. In the Mpro protein, a stable conformation was found after 40 ns; for the RdRp protein, a stable pocket was found from 20–75 ns; and for the ACE2 protein, a stable conformation was reached after 12 ns (Figure 7A). We plot the RMSF (root mean square fluctuation) for the complex ACE2-TODC-3M and Spike-TODC-3M to identify possible residues that may or may not facilitate the ligand binding stability (Figure S4). In both complexes, the key residues of the DP (Table S1) had very little conformational change, indicating low flexibility. The changes for the ACE2-TODC-3M and Spike-TODC-3M were around 0.2 nm and 0.15 nm, respectively. The PCA indicated overall expansion of the complex during the 100 ns simulation, where the principal components (PCs) contributed to global motion. Two directional movements were assessed: eigenvector 1 (PC1) and eigenvector 2 (PC2). 

As shown in Figure S5A, dispersion of TODC-3M in the tested proteins was observed, with the most significant conformational change occurring in the Mpro and RdRp proteins. A 2D-FEL plot for the complex was generated using the PC1 and PC2 to analyze the minimum and maximum energies. TODC-3M exhibited a stable conformation with all four proteins, but the ACE2-TODC-3M complex was the least energetically stable (Figure 7B). Clustering analysis was conducted to search for the more representative structure (MRS) during the simulation. In the ACE2-TODC-3M and Spike-TODC-3M complexes, the ligand remained close to the initial docking position (DP) pocket (Figure 7C,F); for the Mpro-TODC-3M and RdRp-TODC-3M complexes, the ligand moved to a more stable pocket (Figure 7D,E).

**Figure 8:**
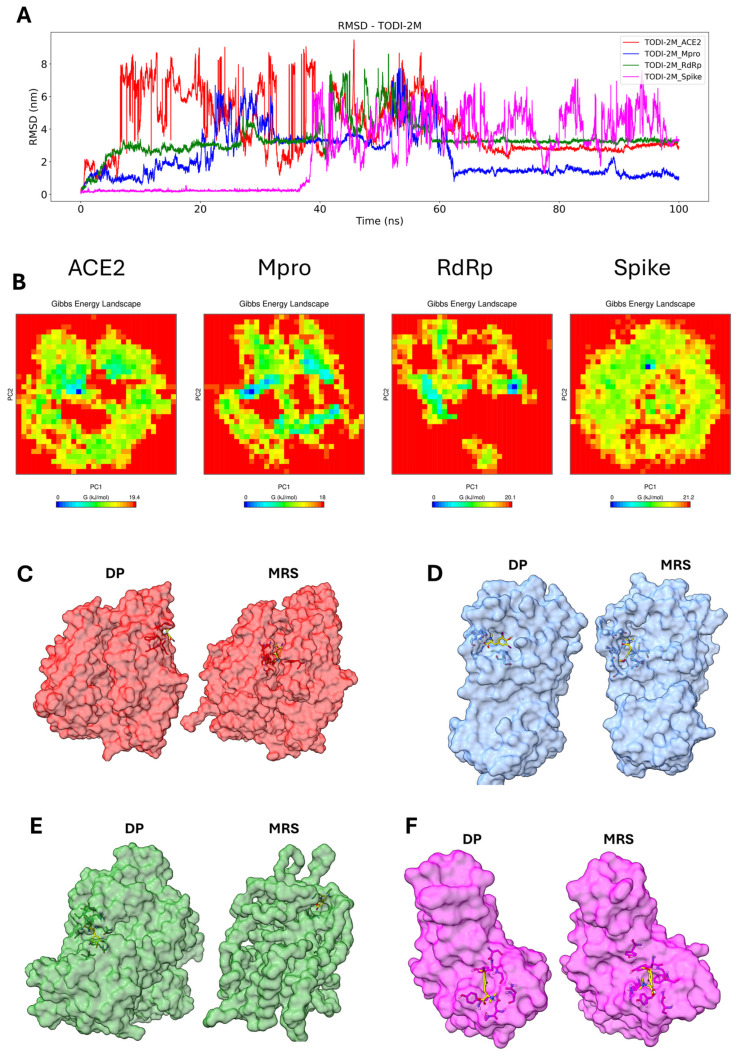
Molecular dynamics analysis of the TODI-2M compound with the target proteins. RMSD from the molecular dynamic trajectories for TODI-2M, ACE2 (red), Mpro (blue), RdRp (green), and spike (magenta) (**A**). PCA-based FEL of the complex; blue indicates the minimum energy (stable conformation), and red indicates the maximum energy (unstable conformation) (**B**). Clustering of the more representative conformations during 100 ns: DP, MRS, ACE2 (**C**), Mpro (**D**), RdRp (**E**), and spike (**F**). TODI-2M is represented in yellow.

The initial conformation (DP) of the TODI-2M compound was preserved with the spike protein only during the first 38 ns. The initial conformation of the other proteins was lost within the first ns of the simulation, and other pockets with more stable interactions were found. For the Mpro protein, a stable conformation was observed after 62 ns; for the RdRp protein, a stable pocket was observed after 60 ns; and for the ACE2 protein, a stable conformation was observed after 65 ns (Figure 8A). Figure S5B shows the instability of the TODI-2M ligand interaction with the tested proteins due to dispersion in PC1 and PC2. TODI-2M displayed a few stable conformations with all four proteins (Figure 8B). Clustering analysis revealed a change in the DP pocket for the ACE2-TODI-2M and RdRp-TODI-2M complexes (Figure 8C,E), whereas it remained close to the DP pocket in the Mpro-TODI-2M and Spike-TODI-2M complexes (Figure 8D,F).

**Figure 9:**
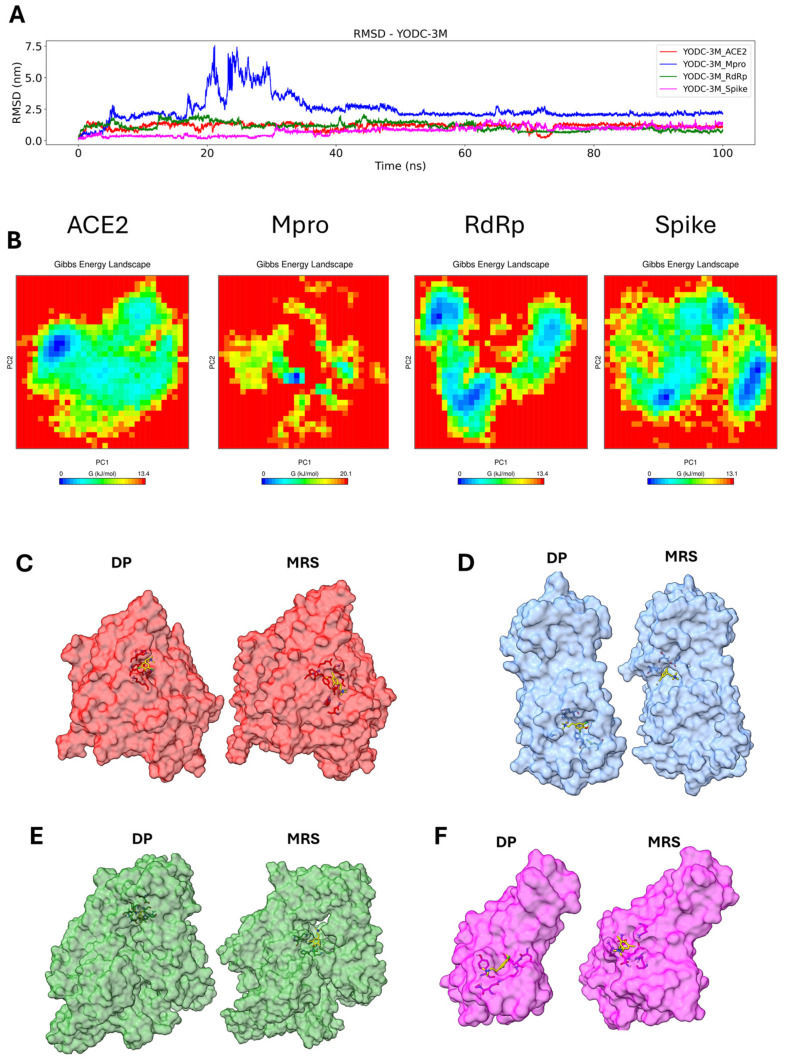
Molecular dynamics analysis of the YODC-3M compound with the target proteins. RMSD from the molecular dynamic trajectories for YODC-3M, ACE2 (red), Mpro (blue), RdRp (green), and spike (magenta) (**A**). PCA-based FEL of the complex; blue indicates the minimum energy (stable conformation), and red indicates the maximum energy (unstable conformation) (**B**). Clustering of the more representative conformations during 100 ns: DP, MRS, ACE2 (**C**), Mpro (**D**), RdRp (**E**), and spike (**F**). YODC-3M is represented in yellow.

The YODC-3M compound showed the most stable interactions during the 100 ns of simulation with the ACE2, Mpro, and RdRp proteins, leaving the initial docking conformation and finding a more stable pocket in these proteins. With the spike protein, the ligand maintained a position close to the initial docking position, showing a stable interaction in the docking pocket. In the Mpro protein, a stable conformation was found after 35 ns, and stable RdRp and ACE2 pockets were identified in the first nanoseconds of the simulation (Figure 9A). Figure S5C shows the instability of the YODC-3M ligand interaction with the Mpro protein due to dispersion in PC1 and PC2. YODC-3M displayed stable conformations with the ACE2, RdRp, and spike proteins (Figure 9B). Clustering analysis revealed a change in the DP pocket for the Mpro-YODC-3M and RdRp-YODC-3M complexes (Figure 9C,F), which remained close to the DP pocket in the ACE2-YODC-3M and Spike-YODC-3M complexes (Figure 9D,F).

## 3. Discussion

Although the WHO declared the end of the COVID-19 pandemic in 2023 [30], this disease remains a public health problem, and few effective treatments are available for the entire population. Our study evaluated 15 synthetic dihalogenated compounds derived from L-tyrosine as antiviral candidates against SARS-CoV-2.

The results of the cytotoxicity assays in Vero-E6 cells revealed that most of these compounds were not toxic at concentrations ranging from 9.4–300 µM, except for TDB-3M, TODB-2M, TODI-2M, and YDB-3M (Figure 2). Similarly, synthetic halotyrosines dihalogenated with bromine and chlorine, as evaluated by Loaiza-Cano et al., did not show toxicity in U937 cells (promonocytic macrophage line) at a concentration of 250 µM [24]. Moreover, those dihalogenated with bromine and chlorine and derivatives of L-tyrosine compounds did not have significant cytotoxic effects on the TZM-bl cell line at concentrations of 9.4–300 µM, except for YDB-3M, similar to the results obtained in our study [25]. Tian et al. evaluated the cytotoxicity of halogenated tyrosine with chlorine, bromine, or iodine in Chinese hamster ovary cells (CHO-K1-cell line) and reported that halogenated compounds with iodine showed more significant cytotoxicity than the other groups did [31]. However, our study revealed that among the five compounds dihalogenated with iodine, only TODI-2M was cytotoxic to Vero-E6 cells. This suggests that the cytotoxicity of the halogenated compounds derived from L-tyrosine is low but depends on the cell type evaluated and the structural modifications of the molecules.

On the other hand, similar to previous reports [25], our in silico toxicity analysis revealed that the 15 dihalogenated compounds had a low probability of biological toxicity (ADMET_Risk score < 2.9), with values even lower than those given to the reference drugs. As shown in Table 1, the compounds TODC-3M, TODI-2M, and YODC-3M were not associated with hepatotoxicity (increasing levels of liver enzymes such as SGOT, SGPT, ALP, and LDH). The compounds with the lowest risk scores were TODI-2M and YODB-3M. Our findings concerning toxicity suggest that dihalogenated compounds could be safe for humans. However, in vivo studies are needed to confirm this hypothesis.

With respect to antiviral activity, YDB-3M, YDI-3M, TODC-3M, TODI-2M, and YODC-3M inhibited SARS-CoV-2 by a combined strategy, the last three being the promising compounds (Figure 3). The antiviral effects of halogenated compounds have been previously demonstrated against a wide range of viruses [24,25,32]. Some antiviral mechanisms suggested for these compounds are the inhibition of early- and post-entry steps of viral replication, such as replication of the viral genome and viral protein synthesis [24,25].

One of the strongest hypotheses suggests that compounds derived from L-tyrosine can induce endoplasmic reticulum (ER) stress [33,34]. This effect, consequently, could interfere with some stages of the viral replication cycle, in which this organelle is involved, such as the formation of the replication–transcription complex (RTC), viral protein folding, assembly, maturation, and release of the SARS-CoV-2 virion [35,36]. Furthermore, unfolded/misfolded proteins can accumulate in the ER and initiate an unfolded protein response (UPR), leading to the activation of autophagy [33], a process that can induce the sequestration of structural proteins or even full viral particles within autophagosomes to be subsequently degraded by lysosomal enzymes [37]. Considering that another ER stress inducer, thapsigargin, efficiently inhibits SARS-CoV-2 replication [38] and that our compounds are also potential inducers of this condition, we hypothesize that this could be a possible explanation for the antiviral effect observed in this study. However, more studies are necessary to clarify this issue.

Previously, Serna et al. reported that different dihalogenated derivatives of L-tyrosine inhibited HIV-1 replication [25] at low concentrations. Similarly, our study revealed that TODC-3M and YODC-3M reduced the viral titer of SARS-CoV-2 to the lowest concentration evaluated (Figure 3). This may be due to the formation of non-visible aggregates, precipitates of the compound, or saturation of the cell with it, for which antiviral activity is not observed at higher concentrations [39,40,41]. Evaluating additional concentrations could help determine the minimum concentration in which these compounds are effective against SARS-CoV-2.

As previously proposed for other viruses [24,25], TODC-3M and TODI-2M exhibited antiviral potential against SARS-CoV-2 in stages after the virus entered the cell (Figure 4). Accordingly, we measured the replication of the viral genome to identify a possible antiviral mechanism, revealing that both compounds induced a significant reduction in E gene RNA (Figure 5). Similarly, a study reported that compounds derived from L-tyrosine and dihalogenated with bromine inhibited the RNA of CHIKV, a virus that shares the same genomic nature as SARS-CoV-2 [24]. Therefore, we suggest that the altered release of infectious viral particles could be due to a reduction in genome replication (Figure 6).

**Figure 10:**
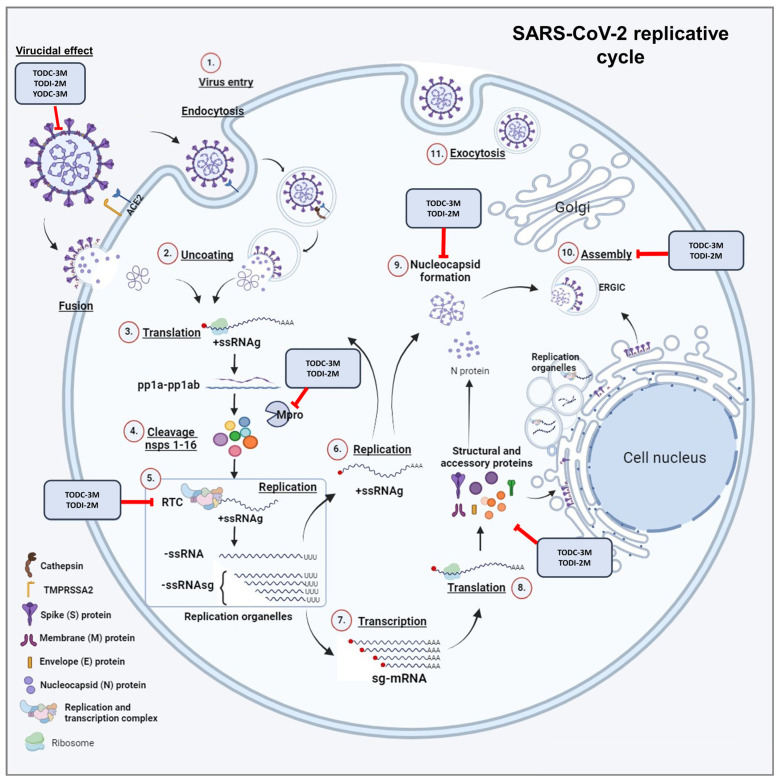
Possible mechanism of the antiviral action of promising compounds against SARS-CoV-2. Halogenated compounds as L-tyrosine derivatives may exert a multimodal action that deserves further investigation. Based on our results, we propose some hypothesis about their antiviral mechanisms against SARS-CoV-2: The compounds TODC-3M, YODC-3M, and TODI-2M exhibited a potential virucidal effect that could be due to the interaction with RBD of spike protein and, consequently, interfere with the viral entry into cells. Additionally, TODC-3M and TODI-2M demonstrated the potential to inhibit later stages of viral entry, which could be related to interference with viral genome replication and/or other downstream steps such as translation, assembly, or release of infectious viral particles. According to in silico analysis, some proposed viral targets of these compounds that could be considered for additional in vitro and in vivo studies are the non-structural proteins Mpro and RdRp of SARS-CoV-2.

These findings could be related to other studies in which compounds derived from L-tyrosine showed favorable affinities with non-structural proteins of other viruses, such as HIV-1, ZIKV, and CHIKV [24,25,26]. Regarding this, our in silico results showed that the TODC-3M and TODI-2M ligands have favorable affinities for the viral proteins RdRp and Mpro (Figure 6). In addition, the stability and conformational changes of these complexes, as evaluated by molecular dynamics, revealed that TODC-3M was more stable than the TODI-2M across two proteins and the docking position pocket was used as the starting point of the simulations (Figure 7, Figure 8 and Figure 9). Based on our in vitro and in silico findings, we suggest that an antiviral mechanism for TODC-3M and TODI-2M could be the interaction with RdRp and Mpro, which would affect viral genome replication or processing of viral polyproteins (Figure 10) and, therefore, the release of infectious viral particles. However, complementary studies are needed to confirm this hypothesis.

We reported that TODC-3M, TODI-2M, and YODC-3M inhibited the release of SARS-CoV-2 infectious particles by co-treatment (Figure 4). As expected, they also reduced the amount of viral RNA through this strategy. These results are consistent with studies demonstrating the virucidal activity of compounds containing chlorine (such as TODC-3M and YODC-3M) and iodine (such as TODI-2M), whose halogens are responsible for the oxidative activity that can damage the viral envelope and prevent it from attaching to cells [42,43]. Considering that different commercial products available are chlorine-/iodine-based and the virucidal potential evidenced in this study, we suggest that they could be tested as oral antiseptics or surface disinfectants. This last use would be recommended mainly for TODI-2M because this compound exhibited lower in vitro selectivity [44] than other compounds using a combined strategy. Likewise, additional structural modifications may be included to improve the selectivity of this compound against SARS-CoV-2.

Concerning the virucidal effect observed in vitro, our in silico tests revealed that TODC-3M, TODI-2M, and YODC-3M presented favorable affinity for the spike protein. In addition, the RMSD and PCA-FEL analyses revealed greater stability for the interaction of the chlorine-based ligands (TODC-3M and YODC-3M) with the spike protein than with the iodine-based ligand (TODI-2M). Clustering analysis indicated that TODC-3M and YODC-3M maintained the interaction with the spike protein in the docking position pocket during the simulation and maintained an average of one hydrogen bond, whereas TODI-2M maintained its position at approximately 40 ns in the simulation close to the docking position (Figure 7, Figure 8 and Figure 9). Accordingly, we hypothesize that TODC-3M and YODC-3M could interact with the spike protein, affecting its ability to bind with the cell receptor, and avoid subsequent viral infection (Figure 10). However, other studies must confirm this mechanism. 

Although conventional MD simulation used in this study has limitations in time scale and overcomes high energy levels, this can bring useful information on the interaction in the protein–ligand complex to understand the stability and affinity of the system. Complementary studies to the MD data could be conducted to visit more conformational change with different metastable states separated by large kinetic barriers. These rare events can be explored with methodologies like metadynamics [45], weighted ensemble [46], or gaussian-accelerated MD [47], which permit crossing these barriers to study new states, bring more information about the protein–ligand interaction, find another stable pocket of interaction for the ligands, and explore different possible mechanisms of actions.

Concerning structural differences of promising compounds, TODC-3M and YODC-3M both feature a positively charged nitrogen atom (quaternary amine), whereas TODI-2M contains a ternary amine. This structural difference may enhance molecular interactions through the potential formation of electrostatic bonds, as supported by the docking results for YODC-3M in Figure 6E. Additionally, TODC-3M and TODI-2M possess a carboxyl group, which is absent in YODC-3M. This functional group facilitates the formation of hydrogen bonds, as illustrated in Figure 6B–D. A notable structural feature shared by the promising compounds is the presence of an aromatic ring substituted with a para-methoxyl moiety. However, the structure–activity relationship remains unclear, as the three compounds only partially share structural elements. Further studies are necessary to elucidate the specific contributions of each chemical substitution to the inhibition of viral proteins.

The main limitation of this study was the amount of compound, which prevented us from evaluating more recently circulating variants and other cell lines. Considering the amino acid differences in the spike protein of SARS-CoV-2 variants [48] compared to the ancestral strain, it is expected that the antiviral potential will change, especially against stages of the replicative cycle that involve this protein, such as adhesion or the virucidal effect. For this reason, promising compounds and others with structural changes will be synthesized to improve their efficacy and selectivity, and subsequently, they will be evaluated against the Omicron variant.

## 4. Materials and Methods

### 4.1. Halogenated Compounds Derived from L-Tyrosine

Two groups of halogenated compounds derived from L-tyrosine, structural analogs of molecules extracted from marine sponges, were evaluated. In total, 15 compounds, nine halotyrosines and six halotyramines, were identified. The classification of these compounds is shown in Figure 1.

The dihalogenation of the fifteen compounds was performed by substitution of the phenyl ring at positions 3 and 5.

All the compounds were dissolved in a water solution with 5% dimethyl sulfoxide (DMSO) (Sigma-Aldrich, St. Louis, MO, USA) at a concentration of 1 mg/mL for the preparation of a stock in concentrations dependent on the molecular weight of each compound (according to its chemical nature). The working dilutions were performed in Dulbecco′s modified Eagle′s medium with high glucose (DMEM) (Sigma-Aldrich, St. Louis, MO, USA) at 9.7–300 µM.

All the synthesized compounds were analyzed with nuclear magnetic resonance and high-resolution mass spectrometry. These compounds had purities higher than 98% [25].

### 4.2. Cell Maintenance and Viral Stock

Vero-E6 cells (African green monkey renal epithelium) were purchased from American Type Culture Collection (ATCC). These cells were used for cytotoxicity and antiviral evaluations and were quantified by a plaque assay. The cultures were incubated at 37 °C with 5% CO_2_ in DMEM supplemented with 2% heat-inactivated fetal bovine serum (FBS) (Sigma-Aldrich, St. Louis, MO, USA). 

The infections were performed with an isolation of an early strain of SARS-CoV-2 (lineage B.1) from the University of Antioquia (Medellin, Colombia) [49].

### 4.3. Determination of Cytotoxicity

The in vitro cytotoxic effects of the dihalogenated compounds on Vero-E6 cells were determined by the MTT assay (Sigma-Aldrich, St. Louis, MO, USA). Briefly, Vero-E6 cells were seeded in 96-well plates for 24 h. Then, the cells were treated with serial twofold dilutions of the compounds (9.37 to 300 μM). After 48 h, the cells were washed twice with 1X PBS (phosphate-buffered saline), and an MTT solution (3 mg/mL) was added for 2 h. Later, DMSO was used to dissolve the formed crystals. The absorbance was read at 570 nm in a microplate spectrophotometer (Thermo Scientific™ Multiskan™ GO. Thermo Fisher Scientific Inc. Vantaa, Finland). Each experimental condition was evaluated in quadruplicate in two independent experiments (n = 8). The percentage of viability was calculated from the absorbance of the control cells without treatment (control for cell viability).

In accordance with the ISO 10993-5:2009 standard [50], concentrations of the compounds with cell viability >80% were chosen for the antiviral activity tests.

### 4.4. Evaluation of the Antiviral Activity of Compounds Against SARS-CoV-2 Using Four Treatment Strategies

Four non-cytotoxic concentrations of each compound were evaluated against SARS-CoV-2 in Vero-E6 cells using four antiviral strategies. Initially, a combined strategy (pre- and post-treatment) was used to evaluate 15 halogenated compounds. Briefly, cells seeded in 96-well plates were incubated with 50 μL of the compounds for 1 h (pre-treatment); then, the viral inoculum (MOI of 0.01) was removed, the monolayer was added, and the cells were incubated for 1 h to be infected. Then, the inoculum was removed, 150 μL of the compounds was added again (post-treatment), and the cells were incubated for 48 h. The supernatants were subsequently collected and stored at −80 °C until subsequent quantification of the infectious viral particles by a plaque assay. Four replicates and two experimental units of each compound were used (n = 8). CQ at 50 μM was used as a positive control of viral inhibition. A control for infection without treatment (untreated control) was used to calculate the percent inhibition.

The compounds that resulted in a >40% reduction in viral replication (promising compounds) when the combined strategy was used were evaluated through three individual treatment strategies (pre-, post-, and co-treatment). In the pre-treatment strategy, the Vero-E6 cells were incubated with the test compounds for 1 h. The treatment mixture was removed, infection (MOI of 0.01) was carried out for 1 h, the inoculum was removed, and the cells were incubated for 48 h in DMEM supplemented with 2% FBS. For the post-treatment strategy, the cells were incubated with the compounds for 48 h after the viral infection. In the co-treatment strategy, the compound was incubated with the virus for one hour at 4 °C; then, this mixture was added to the cells, which were incubated for one hour to allow infection. The number of viral particles in the collected supernatants was quantified by a plaque assay, and the amount of viral RNA in the treated monolayers was quantified by qRT-PCR.

### 4.5. Quantification of Viral Particles by Plaque Assay

The plaque assay was carried out on Vero-E6 cells previously seeded in 24-well dishes. Serial dilutions of supernatants collected from the antiviral activity assays were added to the cell monolayers and incubated for 1.5 h. Subsequently, the inoculum was removed, and 1.5 mL of semisolid medium (2% carboxymethylcellulose, 1X DMEM, 2% SBF, 1% antibiotic/antimycotic) was added to each well. The cells were incubated for 5 days at 37 °C until plaque formation was evident.

After incubation, the medium was removed, and the cells were washed twice with 1X PBS. The cells were subsequently incubated for 30 min with a mixture of 4% formaldehyde/1% crystal violet to fix and stain. Later, the dye was removed, and the samples were washed with PBS. Finally, the number of plaques was counted, and the percentage of plaque inhibition was calculated by comparing the viral titer of the cells treated with the control of infection without treatment (untreated control). Two independent experiments with two replicates each were conducted (n = 4).

### 4.6. Viral Quantification by Real-Time RT-PCR

Viral RNA was extracted from monolayers infected with promising compounds through individual antiviral strategies (pre-, post-, and co-treatment strategy).

The RNA was extracted from cells taken on a Zymo DNA/RNA shield using the SaMag viral nucleic acid extraction kit in an automated SaMag-12 nucleic acid extractor (Sacace Biotechnologies). The viral RNA was subsequently amplified using the Luna® Universal Probe One-Step RT-qPCR Kit (New England Biolabs, Ipswich, MA, USA). This kit includes oligonucleotides and a probe to amplify the E gene of SARS-CoV-2. The assay was carried out according to the Berlin protocol for real-time RT-qPCR [51]. RT-qPCR was conducted in a CFX-96 Bio-Rad thermal cycler (Bio-Rad, Hercules, CA, USA). For each strategy, two independent experiments were conducted in duplicate.

The number of viral RNA copies was calculated by extrapolating the cycle at which samples with viral RNA cross the fluorescence threshold (Ct) in a standard curve [52] were previously constructed with a serially diluted plasmid (3180 bp) containing the E gene at a concentration of 2 × 10^9^ copies/μL (plasmid donated by Dr. Jaime Castellanos from Universidad del Bosque [Bogotá, Colombia]). The inhibition percentages were obtained by comparing the viral RNA from monolayers treated with each compound with that from monolayers infected without treatment (untreated control).

### 4.7. In Silico Toxicological Modeling

In silico toxicological modeling of halogenated molecules derived from L-tyrosine was performed using ADMET Predictor® v8 software from Simulation Plus [53], which predicts toxicity parameters in different biological models.

For this purpose, the structure of the molecules was modeled in the ACD/ChemSketch ®12.01 (Freeware Version) program, and the geometries of all the ligands were optimized using Avogadro software (open-source molecular builder and visualization tool) (Version 1.2, Avogadro Chemistry, San Diego, CA, USA) in the lowest energy conformation using the Merck molecular force field 94 (MMFF94) [54,55]. Subsequently, these structures were inputted into ADMET Predictor® v8 software in “mol” format, which was used to perform probabilistic analysis and determine the parameters of interest defined in the study.

On the basis of these probabilistic estimates, the ADMET predictor yields a toxicological risk score between 0 and 7, where 7 is a prediction of a high probability of toxicity in a certain compound that can generate toxicity in different organs or tissues.

### 4.8. Evaluation of the Interactions of Halogenated Compounds with Viral and Cellular Proteins by Molecular Docking

#### 4.8.1. Ligand Preparation

The structure of the halogenated ligands derived from L-tyrosine was built and optimized as described above. The structure of the control ligands was downloaded from the MolView website (https://molview.org/. Access date: 21 April 2024). Then, the charges and degrees of torsion were added for molecular docking in the Autodock tools (ADT) program using the Gasteiger method [56].

#### 4.8.2. Protein Preparation

For molecular docking, three-dimensional structures of three viral proteins (one structural and two non-structural) were downloaded from the Protein Data Bank (PDB), and structures with a resolution equal to or less than 2.5 Å were considered. The three SARS-CoV-2 viral proteins used for analysis were the S protein (PDB code 6M0J), RdRp (PDB: 7BV2), and Mpro (PDB: 6M0K). The cellular protein used was ACE2 (PDB:6M0J).

The 3D models of interest were prepared for docking using UCSF Chimera software, version 1.19 (UCSF Resource for Biocomputing, Visualization, and Informatics, San Francisco, CA, USA) [57], removing water molecules and co-crystallized molecules that were not part of the target protein and ADT, and adding Gasteiger charges and non-polar hydrogens [56].

A bibliographic review was conducted to determine the amino acids in the RdRp and Mpro active sites and the amino acids involved in the binding of S to the cell receptor (RBD, receptor-binding domain). On this basis, box coordinates in the ADT were selected with a spacing of 1 Å and box sizes in the x-, y-, and z-axes between 10 and 48. An exhaustiveness of 20 was used.

#### 4.8.3. Protein–Ligand Interactions

Finally, to determine the best interactions between the viral proteins and the 15 ligands, AutoDock Vina software, version 1.1.2 (Scripps Research Institute, San Diego, CA, USA) [58] was used. Ligands with lower binding energies (less than 0 Kcal/mol) were considered more favorable [59].

The possible interactions were evaluated with Discovery Studio software, version 3.0 (Dassault Systèmes, Paris, France) [60]. Table S1 shows the viral proteins evaluated in the study and the amino acids used as targets for molecular docking.

### 4.9. Molecular Dynamics

The structures of spike, Mpro, RdRb, and ACE2 bound to the best predicted ligand from the molecular docking analyses were subjected to molecular dynamics (MD) simulations of 100 ns, using a dodecahedron box of water surrounding the protein at 310 K. The structures were previously minimized and subjected to NVT/NPT equilibration phases with GROMACS free software, version 2.1 [61]. The CHARMM36 force field [62], a modified Berendsen thermostat [63], and the Parrinello–Rahman barostat [64] were used during the MD phases. The complex was solvated with periodic boundary conditions at a distance of at least 10 Å, and counterions were included in the solvent to make the box neutral. The particle mesh Ewald (PME) method was used to calculate electrostatic interactions with a 1.0 nm short-range threshold. The timestep in MD production runs is every 2 femtoseconds (fs). Because the ligands are not part of the selected CHARMM36 force field, the server CGenFF parameterized the molecules for the MD analysis [62].

The analysis of molecular dynamics was conducted using the commands of the GROMACS software. For the RMSD analysis, we use the command gmx rms; for the RMSF analysis, we use gmx rmsf; for the PCA, we use gmx covar, gmx anaeig; for clustering, we use the gmx cluster; and for free energy landscape, we use gmx sham.

### 4.10. Analysis of Data

#### 4.10.1. Multivariate Analysis

This clustering analysis was performed through a dendrogram (graphic representation) using the nearest neighbor method in Statgraphics v19 (The Plains, VA, USA). In this method, all compounds and commercial drugs are related to finding the compounds most similar to commercial drugs. For this purpose, the in vitro effectiveness (IC50), cytotoxicity (CC50), and in silico toxicity risk (ADMET risk score) of all the compounds were considered.

#### 4.10.2. Statistical Analysis

The results of the experiments are expressed as the means ± SDs or as medians and interquartile ranges of at least two independent experiments. Differences between groups were analyzed by parametric or nonparametric tests according to the distribution of the data. Statistical significance with a *p*-value less than 0.05 was considered for all the cases.

The CC50 corresponds to the concentration of each compound that can reduce cell viability to 50% of that of the control untreated cells, and the IC50 is the concentration that can reduce viral infection to 50% of that of the control infected cells not treated with the compounds. The therapeutic SI was calculated by dividing the CC50 and the IC50, considering an SI > 10 as the best selectivity index [44]. The analysis was performed in GraphPad Prism software v.8.0.1 (San Diego, CA, USA) using a nonlinear regression (fit curve).

## 5. Conclusions

Our study demonstrated the antiviral activity of five dihalogenated compounds derived from L-tyrosine, highlighting three of them, TODC-3M, TODI-2M, and YODC-3M. These compounds showed low toxicity in vitro and in silico, antiviral potential through different antiviral strategies, and favorable affinities with viral proteins according to molecular docking. Despite the low selectivity of TODI-2M, this compound could be considered for the development of a surface disinfectant. 

In general, our in vitro data suggest that the effectiveness of molecules with antiviral activity is not related to the type of halogenation, nor is there a clear pattern or behavior according to their structural modification. In addition, structural changes between tyrosines and tyramines and different numbers of methylations and halogenations did not significantly influence the affinity of the molecules for viral proteins in the in silico analysis.

## Figures and Tables

**Table 1:** In silico toxicity of halotyrosine and halotyramine derivatives in different organs and tissues (ADMET predictor software).

	TDB-3M	TDC-3M	TDI-3M	TODB-3M	TODC-3M	TODI-3M	TODB-2M	TODC-2M	TODI-2M	YDB-3M	YDC-3M	YDI-3M	YODB-3M	YODC-3M	YODI-3M	HCQ	CQ	Molnupiravir	Nirmatrelvir	Remdesivir
Skin sensitization	1	0	0	1	0	0	1	0	0	1	0	0	1	0	0	1	1	0	0	0
Respiratory sensitization	0	0	0	0	0	0	1	1	0	1	1	0	1	1	1	0	0	0	0	0
Endocrine toxicity (estrogen receptor)	1	1	1	1	1	1	1	1	0	1	1	1	0	0	0	0	0	0	0	0
Endocrine toxicity (androgen receptor)	0	0	0	1	1	0	1	1	1	1	1	1	1	1	1	1	1	0	0	0
Cardiac toxicity (hERG K+ channels)	0	0	0	0	0	0	0	0	0	0	0	0	1	1	1	1	1	0	0	0
Chromosomal aberrations	1	1	0	1	1	0	0	0	0	1	1	0	0	0	0	0	0	1	1	0
Neuronal toxicity (phospholipidosis)	0	0	0	0	0	0	0	0	0	0	0	0	1	1	1	1	1	0	0	0
Reproductive toxicity	0	0	0	0	0	0	0	0	1	0	0	1	0	0	0	0	0	0	1	0
ALP increase	0	0	0	0	0	0	0	0	0	0	1	0	0	0	0	0	0	1	1	1
GGT Increase	0	0	0	0	0	0	1	0	1	0	0	0	0	0	0	0	0	1	0	0
LDH increase	0	0	0	0	0	0	0	0	0	0	1	0	1	0	0	0	0	1	0	1
SGOT increase	0	0	0	0	0	0	0	0	0	0	0	0	0	0	0	0	0	0	0	0
SGPT increase	0	0	0	0	0	0	0	0	0	0	0	0	0	0	0	0	0	1	1	0
**ADMET Risk (0–7)**	**1**	**1**	**1.5**	**2**	**1**	**1.8**	**1**	**1.5**	**0.5**	**1.6**	**2.4**	**1.6**	**0.1**	**1.9**	**1**	**4.8**	**4.4**	**3.7**	**5.5**	**5.1**

ALP: alkaline phosphatase, GGT: gamma-glutamyl transferase, LDH: lactate dehydrogenase, SGOT: aspartate aminotransferase, SGPT: alanine aminotransferase. HCQ: hydroxychloroquine. CQ: Chloroquine. 1: probably toxic, and 0: probably non-toxic.

**Table 2:** Promising compounds with antiviral activity against the lineage B.1 of SARS-CoV-2: Results of in vitro and in silico assays.

	In Vitro				In Silico				
Ligands	Cytotoxicity	Antiviral Activity by Combined Strategy		Selectivity	Molecular Docking (Kcal/mol)				Toxicity
	CC50 (uM)	Inhibition * (%)	IC50 (μM)	SI	Spike	RdRp	Mpro	ACE2	ADMET Risk
TODC-3M	3280	73.4%	47.1	69.6	−5.0	−4.9	−5.0	−4.7	1
TODI-2M	281.1	65.7%	90.1	3.1	−4.9	−4.7	−5.2	−4.3	0.5
YODC-3M	~351.1	43.3%	18.8	~18.7	−4.9	−4.3	−4.7	−4.0	1.9

CC50: 50% cytotoxic concentration; IC50: half maximal inhibitory concentration; SI: selectivity index; RdRp: RNA-dependent RNA polymerase; Mpro: main protease; ACE2: angiotensin-converting enzyme 2. * This value corresponds to the maximum percentage of inhibition obtained for the compound by the combined strategy.

## Data Availability

The data that supports the results is confidential but can be made available upon reasonable request from other researchers.

## Supplementary Materials

The following supporting information can be downloaded at: https://www.mdpi.com/article/10.3390/molecules30071419/s1. Figure S1: Vero-E6 cells were treated for 48 h with different concentrations of DMSO (from 0.04% to 5%) (A). Vero-E6 cells were treated with 50 or 100 µM CQ (B); Figure S2: TODC-3M is most similar in terms of effectiveness and toxicity to commercially used drugs; Figure S3: 3D visualization of molecular docking among ligands and viral and cellular proteins. This visualization was created in AutoDock Tools with the information obtained from the molecular docking performed in AutoDock Vina. Complexes Mpro-TODC-3M (A), Spike-TODC-3M (B); Mpro-TODI-2M (C); and Spike-YODC-3M (D); Figure S4: The root-mean-square-fluctuation (RMSF) of the complexes formed between TODC-3M and target proteins. RMSF for the Spike (A) and ACE2 (B) proteins in complex with TODC-3M, in orange, highlight the key residues in the Docking position pocket; Figure S5: Principal component analysis (PCA) of protein–ligand complexes.TODC-3M with ACE2 (red), Mpro (blue), RdRp (green), and Spike (yellow) (A). TODI-2M with ACE2 (red), Mpro (blue), RdRp (green), and Spike (yellow) (B). YODC-3M with ACE2 (red), Mpro (blue), RdRp (green), and Spike (yellow) (C); Table S1: Viral and cellular proteins and their active or binding sites as targets for molecular docking.
