# Supplementary material for: Antiviral Activity of Halogenated Compounds Derived from L-Tyrosine Against SARS-CoV-2
Source: Molecules. 2025 Mar 22;30(7):1419. doi: 10.3390/molecules30071419 (PMC11990460; doi:10.3390/molecules30071419)
Supplement: Supplementary file 1 [file molecules-30-01419-s001.zip › molecules-3469303-supplementary.pdf]

Supplementary material

Supplementary figures

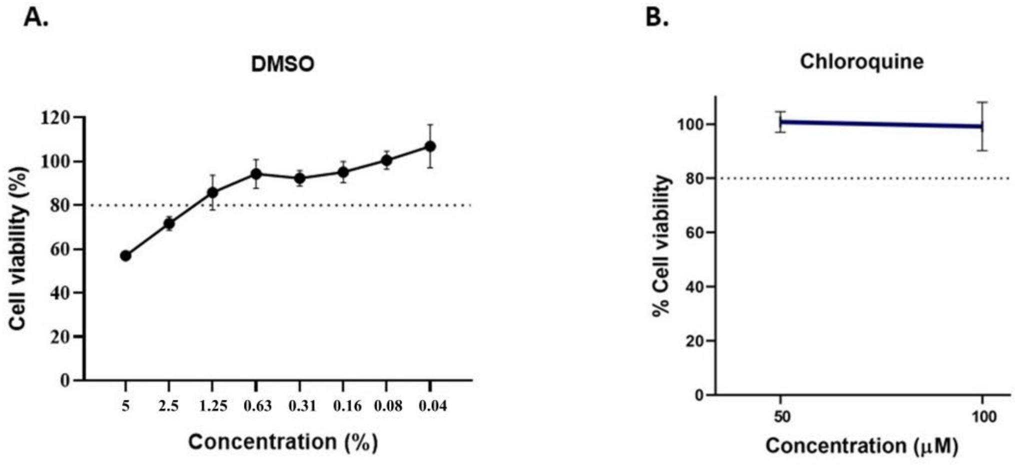

Supplementary Figure S1: Cytotoxicity of the controls in Vero E6 cells. Vero-E6 cells were treated for 48 h with different concentrations of DMSO (from 0.04% to 5%) (**A).** Vero-E6 cells were treated with 50 or 100 μM CQ **(B).** The percentages of cell viability were calculated relative to an untreated control. Each concentration was evaluated in quadruplicate in three independent experiments. The results are shown as the means ± standard deviations. Concentrations that decreased cell viability to less than 80% were considered toxic.

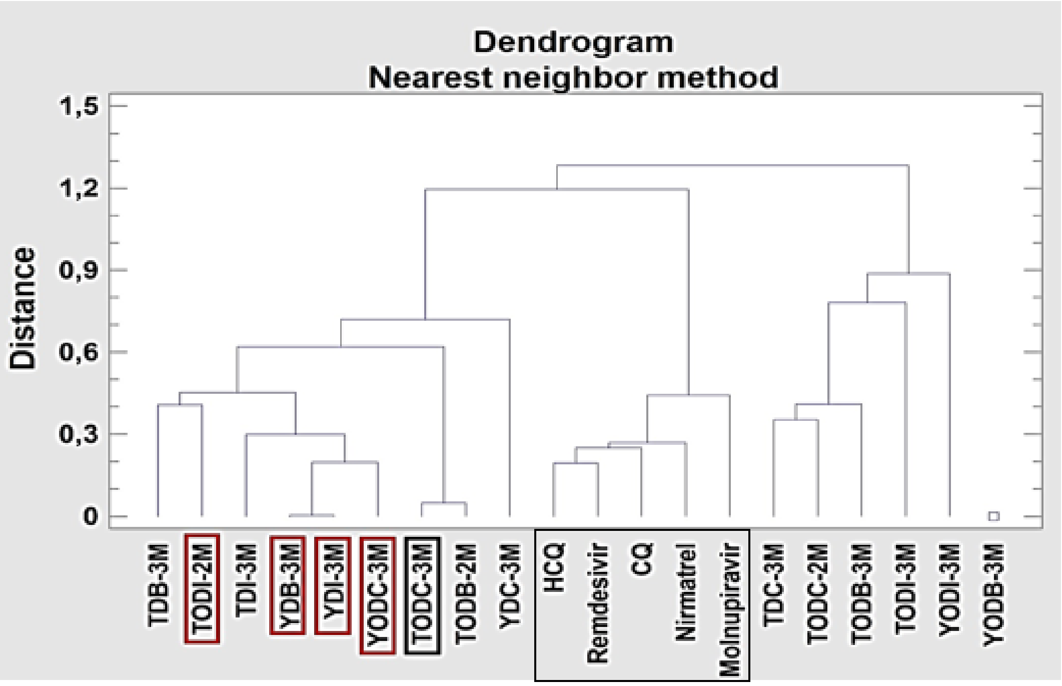

Supplementary Figure S2. TODC-3M is most similar in terms of effectiveness and toxicity to commercially used drugs. Hierarchical cluster analysis was performed by the nearest neighbor method, which is based on the *in vitro* and *in silico* effectiveness and toxicity parameters of the compounds**.** In red: compounds with antiviral activity using the combined strategy; in black: compounds with antiviral activity similar to that of commercially used medications (nearest neighbor) in terms of toxicity and antiviral effectiveness.

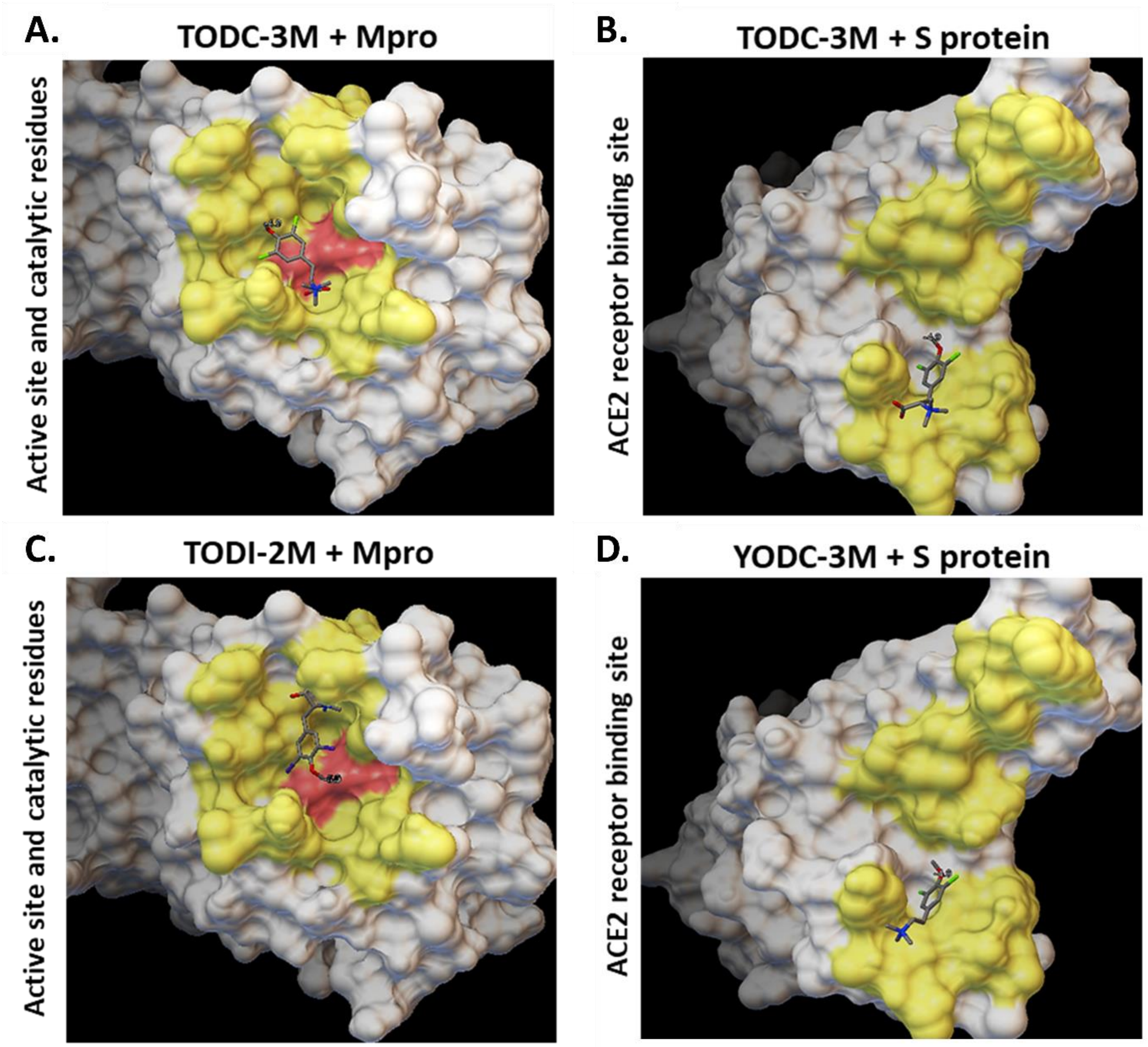

Supplementary Figure S3. 3D visualization of molecular docking among ligands and viral and cellular proteins. This visualization was created in AutoDock Tools with the information obtained from the molecular docking performed in AutoDock Vina. Complexes Mpro-TODC-3M **(A)**, Spike-TODC-3M **(B)**; Mpro-TODI-2M **(C)**; and Spike-YODC-3M **(D)**. The amino acids necessary for binding to the substrate or natural ligand are highlighted in yellow. For the Mpro enzyme, the amino acids of its active site are highlighted in yellow, and the two amino acids that perform its catalytic function are highlighted in pink (His41 and Cys145). The amino acids that interact with the ACE2 receptor are highlighted in yellow for the Spike protein.

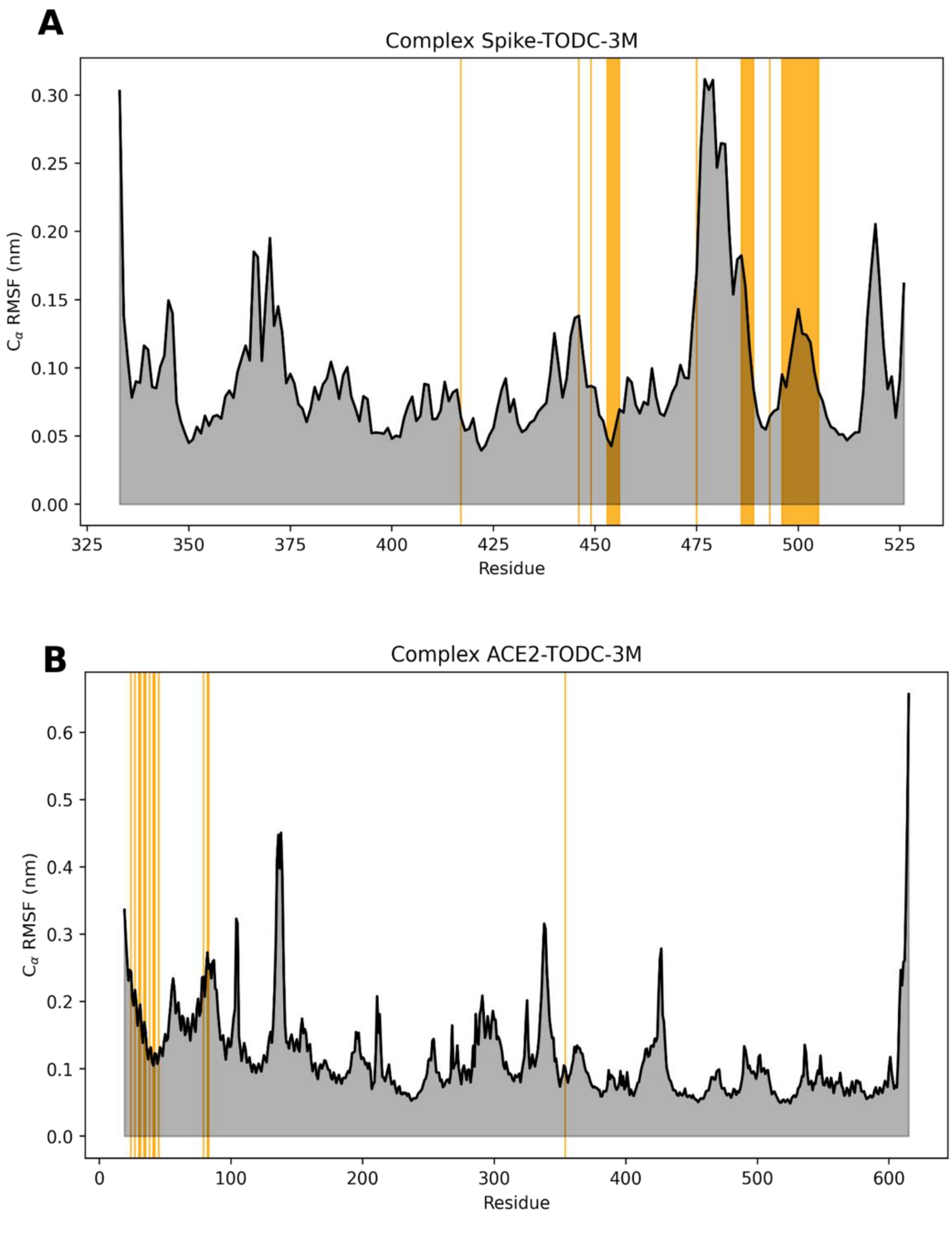

Supplementary Figure S4. The root-mean-square-fluctuation (RMSF) of the complexes formed between TODC-3M and target proteins. RMSF for the Spike **(A)** and ACE2 **(B)** proteins in complex with TODC-3M, in orange, highlight the key residues in the Docking position pocket.

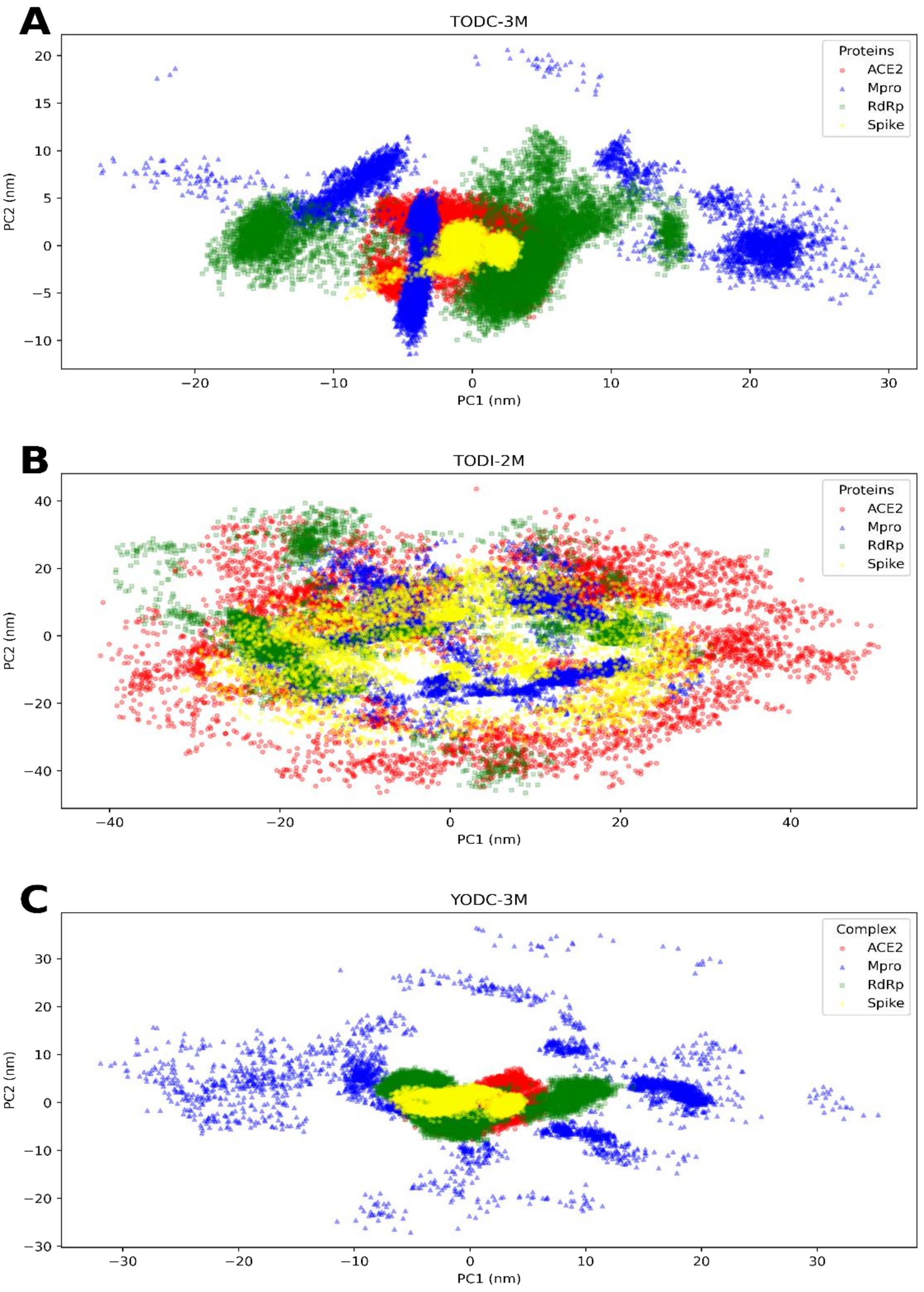

Supplementary Figure S5: Principal component analysis (PCA) of protein–ligand complexes. TODC-3M with ACE2 (red), Mpro (blue), RdRp (green), and Spike (yellow) **(A).** TODI-2M with ACE2 (red), Mpro (blue), RdRp (green), and Spike (yellow) **(B).** YODC-3M with ACE2 (red), Mpro (blue), RdRp (green), and Spike (yellow) **(C).**

Supplementary tables

Supplementary Table S1. Viral and cellular proteins and their active or binding sites as targets for molecular docking.

Protein	PDB code	Resolution (Å)	Active site or binding site amino acids	Coordenates (x,y,z)
**RBD domain, Spike protein (Chain B)**	6M0J	2.45	Lys417, Gly446, Tyr449, Tyr453, Leu455, Phe456, Ala475, Phe486, Asn487, Tyr489, Gln493, Gly496, Gln498, Thr500, Asn501, Gly502 and Tyr505	center x= -37.058, center y= 28.364, center z= 3.838
**RdRp (Chain A)**	7BV2	2.5	Lys593, Trp598, Met601, Thr687, Ala688, Ala691, Ser759, Asp760, Asp761, Cys813, Gln815, Pro830 and Asp618	center x= 90,485, center y= 86,069, center z= 97,835
**Mpro (Chain A)**	6M0K	1.5	Thr24, Thr25, Thr26, Leu27, His41, Cys44, Met49, Tyr54, Phe140, Leu141, Asn142, Gly143, Ser144, Cys145, His163, His164, Met165, Glu166, Leu167, Pro168, His172, Phe181, Phe185, Asp187, Arg188, Gln189, Thr189, Ala190, Ala191 and Gln192	center x= -14.121, center y= 13.96, center z= 68.699
**ACE-2**	6M0J	2.45	Gln24, Thr27, Asp30, Lys31, His34, Glu35, Asp38, Tyr41, Gln42, Leu45, Leu79, Met82, Tyr 83, Gly354 and Lys353	center x= -35.972, center y= 27.583, center z= 2.333

Alanine: Ala, arginine: Arg, asparagine: Asn, aspartic acid: Asp, cysteine: Cys glutamine: Gln, glutamic acid: Glu, glycine: Gly, histidine: His, isoleucine: Ile, leucine: Leu, lysine: Lys, methionine: Met, phenylalanine: Phe, proline: Pro, serine: Ser, threonine: Thr, tryptophan: Trp, tyrosine: Tyr, valine: Val
